# Genotyping-by-Sequencing Strategy for Integrating Genomic Structure, Diversity and Performance of Various Japanese Quail (*Coturnix japonica*) Breeds

**DOI:** 10.3390/ani13223439

**Published:** 2023-11-07

**Authors:** Natalia A. Volkova, Michael N. Romanov, Alexandra S. Abdelmanova, Polina V. Larionova, Nadezhda Yu. German, Anastasia N. Vetokh, Alexey V. Shakhin, Ludmila A. Volkova, Dmitry V. Anshakov, Vladimir I. Fisinin, Valeriy G. Narushin, Darren K. Griffin, Johann Sölkner, Gottfried Brem, John C. McEwan, Rudiger Brauning, Natalia A. Zinovieva

**Affiliations:** 1 L. K. Ernst Federal Research Center for Animal Husbandry, Dubrovitsy, Podolsk 142132, Moscow Oblast, Russia; natavolkova@inbox.ru (N.A.V.); abdelmanova@vij.ru (A.S.A.); volpolina@mail.ru (P.V.L.); ngerman9@gmail.com (N.Y.G.); anastezuya@mail.ru (A.N.V.); alexshahin@mail.ru (A.V.S.); ludavolkova@inbox.ru (L.A.V.); 2 School of Biosciences, University of Kent, Canterbury, Kent CT2 7NJ, UK; d.k.griffin@kent.ac.uk; 3 Breeding and Genetic Center Zagorsk Experimental Breeding Farm—Branch of the Federal Research Centre, All-Russian Poultry Research and Technological Institute, Russian Academy of Sciences, Sergiev Posad 141311, Moscow Oblast, Russia; a89265594669@rambler.ru; 4 Federal Research Center “All-Russian Poultry Research and Technological Institute” of the Russian Academy of Sciences, Sergiev Posad 141311, Moscow Oblast, Russia; olga@vnitip.ru; 5 Research Institute for Environment Treatment, 69032 Zaporizhya, Ukraine; val@vitamarket.com.ua; 6 Vita-Market Co., Ltd., 69032 Zaporizhya, Ukraine; 7 Institute of Livestock Sciences (NUWI), University of Natural Resources and Life Sciences Vienna, 1180 Vienna, Austria; johann.soelkner@boku.ac.at; 8 Institute of Animal Breeding and Genetics, University of Veterinary Medicine, 1210 Vienna, Austria; gottfried.brem@agrobiogen.de; 9 AgResearch, Invermay Agricultural Centre, Mosgiel 9053, New Zealand; john.mcewan@agresearch.co.nz (J.C.M.); rudiger.brauning@agresearch.co.nz (R.B.)

**Keywords:** genotyping-by-sequencing, genetic diversity, genomic structure, phylogeny, performance, Japanese quail, breeds, utility types

## Abstract

**Simple Summary:**

Artificial selection has been applied to domesticated birds for many decades. More recently, this selection has made use of so-called single-nucleotide polymorphism (SNP) markers—simple variants in a DNA sequence. These SNPs can be used for whole-genome screening to detect the unique traces of areas of the genome that are subject to selection. Doing this may help to shed light on the evolutionary and family history (phylogeny) of domestic Japanese quails of different breeds and utility types (e.g., egg, meat or dual-purpose breeds). In this study, 99 birds were used, representing eight breeds (11% of the world’s quail gene pool) and various purposes of use to gather genetic (whole-genome) data in the first-ever analysis of its kind performed on domestic quails. We thereby uncovered evolutionary relationships and points of divergence of individual quail breeds, gleaning important insights into the genetic diversity of domestic quail breeds and their future breeding potential.

**Abstract:**

Traces of long-term artificial selection can be detected in genomes of domesticated birds via whole-genome screening using single-nucleotide polymorphism (SNP) markers. This study thus examined putative genomic regions under selection that are relevant to the development history, divergence and phylogeny among Japanese quails of various breeds and utility types. We sampled 99 birds from eight breeds (11% of the global gene pool) of egg (Japanese, English White, English Black, Tuxedo and Manchurian Golden), meat (Texas White and Pharaoh) and dual-purpose (Estonian) types. The genotyping-by-sequencing analysis was performed for the first time in domestic quails, providing 62,935 SNPs. Using principal component analysis, Neighbor-Net and Admixture algorithms, the studied breeds were characterized according to their genomic architecture, ancestry and direction of selective breeding. Japanese and Pharaoh breeds had the smallest number and length of homozygous segments indicating a lower selective pressure. Tuxedo and Texas White breeds showed the highest values of these indicators and genomic inbreeding suggesting a greater homozygosity. We revealed evidence for the integration of genomic and performance data, and our findings are applicable for elucidating the history of creation and genomic variability in quail breeds that, in turn, will be useful for future breeding improvement strategies.

## 1. Introduction

The study of molecular genetic principles that determine the degree of manifestation of economically significant traits is of crucial importance for increasing agricultural. In so doing, it can be applied to produce the most effective and cost-efficient agricultural products for domestic and world consumption. The poultry industry is one of the key sectors of agricultural production, and its important products are meat and eggs. Poultry meat accounts for 45% of the total global meat production [1] and virtually all egg production. In Russia, the growing market demand for poultry products led, by 2020, to an elevation from 4 to 10% in the share of food products from non-traditional poultry species [2]. An increasing proportion of the world’s egg and meat supply are provided from species of the Phasianidae (pheasant) family [3,4]. Hereby, quail breeding industry products are in special demand worldwide because of the palatability of quail eggs and meat, as well as the early onset of sexual maturity. Because of this, the establishment and growth of large quail farms mean that quail eggs and meat are becoming everyday products [3,4,5,6,7].

The progenitor of contemporary quail breeds, the Japanese quail (*Coturnix japonica* Temminck & Schlegel, 1848), is a migratory bird native to East Asia. Domesticated Japanese quail are a common poultry type used for meat and eggs in Europe, Asia and throughout the world. Quails have been used in genetic research since 1940 [8] and, over time, they have become an increasingly important biological model for developmental, behavioral and biomedical studies [3,4]. Belonging to the same Phasianidae family as chickens, quails have a number of advantages as a research model. They are small, fast growing, and have a short life cycle, reaching sexual maturity in 7–8 weeks after hatching [9]. In comparative biological studies of galliforms, quails show key differences from chickens and some other poultry species, e.g., immune status, migratory and seasonal behavior [3].

Worldwide, there are about 70 domestic Japanese quail breeds or strains, including commercial and laboratory quails [10]. The first Japanese quails were imported into Russia in 1964 for breeding and production purposes. The adult quail population in the former USSR grew steadily year after year, peaking at around 200,000 individual birds [11]. A large collection of quail breeds was first created at the Moscow Timiryazev Agricultural Academy (MTAA) and involved the Pharaoh (PHA), English White (ENW), British Range, Tuxedo (TUX) and Marbled breeds [12] plus a wild-type colored strain developed in the Scientific and Production Association “Complex” (SPAC; Moscow, Russia) by crossing the Marbled and PHA quails. The Marbled breed was created at the MTAA in collaboration with the N.I. Vavilov Institute of General Genetics by subjecting a group of quails to X-rays. A relatively novel dual-purpose Estonian (EST) breed was produced in 1988 by mating the Japanese (JAP), ENW and PHA breeds. According to a cytogenetic analysis, the EST quails can be distinguished from JAP quails by the presence of a centromeric band in autosome 1 that is G-positive and can be utilized as a chromosomal marker for EST (as reviewed in [11]). Another large collection of quail breeds currently exists in the Zagorsk Experimental Breeding Farm, All-Russian Poultry Research and Technological Institute (ZEBF/ARPRTI), and embraces JAP, ENW, English Black (ENB), TUX, Manchurian Golden (MAG), EST, PHA, Texas White (TEW) and a few other quail breeds and strains [13].

To create a competitive breeder stock for quails, it is necessary to use modern methodologies of genetic and genomic analysis aimed at increasing the efficiency of selection and breeding work. For instance, identified sex-linked genes for phenotypic traits, e.g., recessive genes for imperfect albinism (*al*) and brown (*br*), can be employed for autosexing (sex sorting) of newly hatched chicks [10,11,14,15].

The integration of high-throughput, next-generation sequencing-based genomic technologies into practical quail breeding should therefore be carried out, at the initial stage, by assessing the genomic architecture characteristic of a particular breed. A subsequent comparison of the genomic structure of quail breeds of different origin and direction of selective breeding is necessary. This can prove to be an effective approach to identify specific genomic regions that are either related to recent divergence and/or earlier breeding differentiation. In addition, such an investigation facilitates a genome-wide assessment and refinement of the diversity and phylogeny amongst various quail breeds. Genotyping by sequencing (GBS), one of the restriction enzyme-based enrichment approaches designed initially for plants [16], is a promising strategy for reducing the financial burden of selection strategies via high sample multiplexing, focusing the sequenced genome areas on randomly distributed read tags [17]. Being a relatively affordable and widely applicable substitute for concurrent single-nucleotide polymorphism (SNP) mining and genotyping in plants (e.g., [16,18,19]), GBS is also increasingly being used in animals (e.g., [17,19,20,21,22]). Indeed, there was a recent report that GBS has been utilized for evaluating demographic history and genetic divergence in wild African harlequin quail (*Coturnix delegorguei delegorguei*) populations of Kenya [22]. Ravagni et al. [23] employed GBS to explore the evolutionary history of an island endemic, the common quail (*Coturnix coturnix*) in the Azores archipelago. To the best of our knowledge, however, there have been no studies using this technique to characterize the genomic architecture and diversity in the divergently selected breeds of the domesticated Japanese quail.

In the current investigation, we thus aimed to examine and compare the variability and phylogeny among the genomes of eight divergently selected breeds of egg-type, meat-type and dual-purpose quails that represented a significant portion (~11%) of the global gene pool of quail breeds. In accordance with this goal, the GBS approach was applied to characterize the genetic structure of these quail populations. This information is essential for maintaining their genomic diversity and facilitating efficient breeding in the future.

## 2. Materials and Methods

### 2.1. Experimental Birds and Performance Data

Quails were hatched from fertile eggs purchased from the Genofond LLC (ZEBF/ARPRTI; [13]), grown at the L. K. Ernst Federal Research Centre for Animal Husbandry (LKEFRCAH) [24], and sampled for DNA. The following eight quail breeds were used in this experiment (Table 1): JAP, ENW, ENB, TUX, and MAG (of egg type); TEW and PHA (of meat type); and EST (of dual purpose).

For each breed, the number of females (*n*) was taken into account, for which the following performance indicators (as mean ± standard deviation) were collected: egg number (EN) for 180 days from the start of lay; egg weight (EW) obtained at the age of 180 to 210 days (for each female, mean was calculated over all eggs laid during a given period); and body weight (BW) of females at the ages of 6 weeks and 6 months. These data were subsequently assessed and compared with calculations of interbreed genetic variability resulting from the GBS analysis. Herewith, we proposed a hypothesis that a certain degree of “congruence” (or, in other words, integration) between phenotypic and genomic data can take place for this sample of quail breeds. For this purpose, an appropriate mathematical analysis was undertaken using a new index, Narushin’s IPI (Integral Performance Index). The latter was recently established by Vakhrameev et al. [31] to evaluate the main economically important traits (i.e., EN, EW and female BW) in various chicken breeds and was originally designated as EY/W (where EY was the product of mean EN and EW, and W was mean female BW). Here, we renamed this index after the author of that study, Valeriy G. Narushin, who proposed this indicator, and calculated it using the corresponding formula:

$$IPI=\frac{EN\cdot EW}{BW}$$

where EN is egg number, EW is egg weight (in g), and BW is female body weight at 6 months of age (in g), all values being calculated as breed means.

Statistical evaluation of raw performance data and means was performed using Microsoft Excel (version 16.66.1). Student’s *t*-test was implemented to compare the means of breeds in pairwise mode and determine the significance of the differences between them using Microsoft Excel’s T.TEST function and GraphPad online calculator [32].

### 2.2. Sampling and DNA Isolation

Feather samples containing pulp were obtained from 106 quails of all the breeds studied. DNA extraction was performed using the Syntol kit for DNA isolation from animal tissues (Syntol, Moscow, Russia). The DNA solution concentration was determined using a Qubit 3.0 fluorimeter (Thermo Fisher Scientific, Wilmington, DE, USA). To check the purity of the extracted DNA, the OD260/280 ratio was tested using a NanoDrop-2000 instrument (Thermo Fisher Scientific).

### 2.3. Sequencing, Genotyping and Quality Control of SNPs

Quail genotyping was performed using GBS analysis [16] that included the basic steps of library construction, sequencing, sequence quality control (QC), SNP detection, and construction of a genomic relationship matrix. In particular, the methods described in Elshire et al. [16], with changes as in Dodds et al. [33], were implemented to build the GBS libraries. A *Pst*I–*Msp*I double-digest was used to generate one GBS library that also contained negative control samples devoid of DNA. Libraries were subjected to a Pippin Prep (SAGE Science, Beverly, MA, USA) to choose fragments with a size between 220 and 340 bp (genomic sequence plus 148 bp adapters). We employed a set of 768 barcodes designed by Integrated DNA Technologies, Inc. (Coralville, IA, USA) and Illumina (Illumina, Inc., San Diego, CA, USA) that differed from each other by at least three mutational steps. The corresponding adapter sequences were as follows: *Pst*I_Common_F, AGATCGGAAGAGCACACGTCTGAACTCCAGTCAC; *Pst*I_Common_R, GTGACTGGAGTTCAGACGTGTGCTCTTCCGATCTTGCA; *Msp*I(Y)_Common_F, CGAGATCGGAAGAGCGGACTTTAAGC; and *Msp*I_Common_R, GTGACTGGAGTTCAGACGTGTGCTCTTCCGATCT. Single-end sequencing (1 × 101 bp) was performed utilizing a NovaSeq 6000 instrument (Illumina, Inc.) and the appropriate v1.5 reagents. Raw fastq files were quality checked using a custom QC pipeline, DECONVQC [19,34]. As one of the QC steps, raw fastq files were quality tested using FastQC [35].

As a reference genome, we used the Japanese quail genome assembly Coturnix japonica 2.0 [36], along with the databases Ensembl 104 and Ensembl Genomes 51 (released on 7 May 2021; [37]). Removal of adapter sequences and demultiplexing of the fastq file, i.e., its separation by samples to produce individual fastq files using a list of barcodes, were executed using the cutadapt program [38,39]. The QC of fastq files was carried out in the FastQC program [35]. To call SNPs from the GBS data, the bioinformatics workflow snpGBS [40,41] was employed. The bowtie2 package was used to align the individual fastq files to the reference genome and index them [42], while sorting of bam files was performed using samtools [43,44].

Joint genotyping of the resulting files was implemented using bcftools [44] generating one multi sample VCF file. After filtering, 80,673 SNPs were used for subsequent analysis steps. The data was generated into a file format acceptable for further analysis using the R software package [45]. The PLINK 1.9 program [46] was employed to control the quality of SNP detection. The obtained quail genotypes were filtered according to the genotyping efficiency parameter (mind 0.25), and SNPs genotyped in less than 90% samples (geno 0.1) were excluded from the analysis. The final dataset used for the genome-wide analyses was 62,935 SNPs out of original 80,673 SNPs. A total of 106 individuals were initially sequenced and genotyped. After removing two quails that did not belong to their respective breeds according to genetic data, a matrix of 104 birds was subject to subsequent analysis. After pulling out five more quails from the total number due to insufficient information about them with regard to the studied SNPs, a total of 99 individuals was investigated in the experiment.

For some types of analysis, e.g., principal component analysis (PCA), analysis of genetic diversity and divergence, construction of phylogenetic networks, analysis of population structure and analysis of gene flow (migration events), an additional linkage disequilibrium (LD) filter was applied to remove loci for which LD was identified within a 50 Kb sliding window with a step of 5. After using the LD filter, 27,171 SNPs were included in the analysis.

### 2.4. Genetic Diversity Assessment

To determine within-population genetic diversity, PLINK 1.9 software package was used. QC was performed at both the individual and SNP levels using PLINK 1.9. By implementing various parameters of the R package diveRsity [47], we computed values of observed heterozygosity (*H_O_*), expected heterozygosity (*H_E_*), unbiased expected heterozygosity (*_U_H_E_*), rarefied allelic richness (*A_R_*; [48]), coefficient inbreeding (*F*_IS_) and coefficient of inbreeding (*_U_F*_IS_) based on unbiased expected heterozygosity.

### 2.5. PCA, Neighbor-Net and Admixture Procedures

For breed clustering, PCA and calculation of identical-by-state (IBS) distances were performed in PLINK1.9. The degree of genetic differentiation of the studied breeds was estimated based on pairwise *F*_ST_ values. Visualization of PCA results was carried out in the R ggplot2 package [49]. Dendrograms based on IBS distances and pairwise *F*_ST_ distances were plotted using an agglomerative method for constructing phylogenetic networks, i.e., Neighbor-Net in SplitsTree 4 [50]. The software Admixture v1.3 [51] for model-based clustering and computation of the related cross-validation (CV) errors was implemented to analyze ancestral populations and genetic impurities, while the BITE R package [52] was used to visualize these results. The Phantasus web program was also used to perform PCA and hierarchical clustering procedures [53]. Using the online T-REX program [54], the Neighbor-Joining [55] trees showing phylogenetic relationships between breeds were built.

Gene flow (migration) events were analyzed using the TreeMix 1.12 program [56]. The analysis considered from 0 to 5 migrations with 30 iterations per migration event. The optimal number of migrations (1) was determined using the *OptM* R package [57]. The best maximum likelihood tree configuration was determined based on the minimum mean standard error of the residual matrix among all iterations.

The analysis of homozygous genomic segments (runs of homozygosity, ROH) was performed using the detectRUNS R package [58] with the following settings: the minimum number of SNPs was 30, and the minimum length was 0.5 Mb.

## 3. Results

### 3.1. Breed Performance

Information for the three major performance characteristics, according to which the IPI index was computed, is given in Table 2. As can be seen, the two meat-type breeds, PHA and TEW, had the lowest IPI values (roughly 5 if rounded up to integers), the dual-purpose breed, EST, had a slightly higher value (~7), and the five egg-type breeds had greater values (~8 to 12).

**Figure 1:**
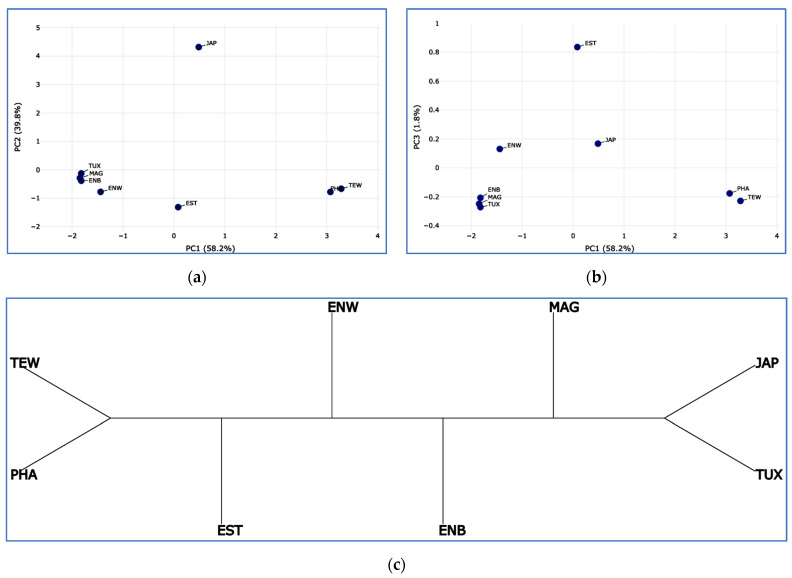
Clustering reconstruction of the eight breeds studied using the IPI-based pairwise Euclidean distances. (**a**,**b**) PCA plots for first (PC1) and second (PC2) components (**a**), and for first (PC1) and third (PC3) components (**b**) using the Phantasus web tool [53]. (**c**) A Neighbor-Joining rootless axial tree built with no proportional edge length and using the Neighbor Joining method [55] and the online T-REX tool [54]. Quail breeds: JAP, Japanese; ENW, English White; ENB, English Black; TUX, Tuxedo; MAG, Manchurian Golden; EST, Estonian; PHA, Pharaoh; TEW, Texas White.

A matrix of interbreed Euclidean distances computed for breed IPI values is presented in Supplementary Table S1. Using it, breed clustering was reconstructed in the form of PCA plots and Neighbor-Joining trees (Figure 1). Notably, a largely similar configuration of breeds was obtained using both clustering techniques, reflecting the breed subdivision into three main types of utility and selection, as well as in accordance with the ranking of IPI values as shown in Table 2.

### 3.2. Analysis of Genetic Diversity

Using around 100 DNA samples from quails of as many different phenotypes as possible, we generated a GBS panel for genotyping the quail breeds. In terms of genetic diversity values (Table 3), TUX quails were characterized by lower values of genetic diversity, as measured by lower levels of expected heterozygosity (*H_E_* = 0.263 vs. the maximum value of 0.310, *p* < 0.001) and allelic richness (*A_R_* = 1.730 vs. the maximum value of 1.864, *p* < 0.001) as compared to the JAP breed. This can be explained by the higher intensity of breeding work in TUX that was aimed at consolidating the desired breed characteristics. The inbreeding coefficient (*F*_IS_) was represented by the maximum value for the JAP population (0.020, with a 95% confidence interval being from 0.016 to 0.024), which may be indicative of a likely growth in gene homozygosity in this population.

Due to the small number of birds in each breed, we also calculated unbiased measures of expected heterozygosity (*_U_H_E_*) and expected inbreeding rate (*_U_F*_IS_) adjusted for small samples. The former was highest in EST (0.313) and JAP (0.319). The latter coefficient was represented by positive values for all breeds ranging from the minimum of 0.011 in TEW to the maximum of 0.046 in JAP quails. High rates of *_U_F*_IS_ were also found in EST (0.032) and MAG (0.031), as well as in the PHA population (0.035). This enabled us to conclude that there was a significantly higher homozygosity of genes in these four populations as compared to other breeds.

### 3.3. Between-Breed Genetic Relationships and Model-Based Clustering

**Figure 2:**
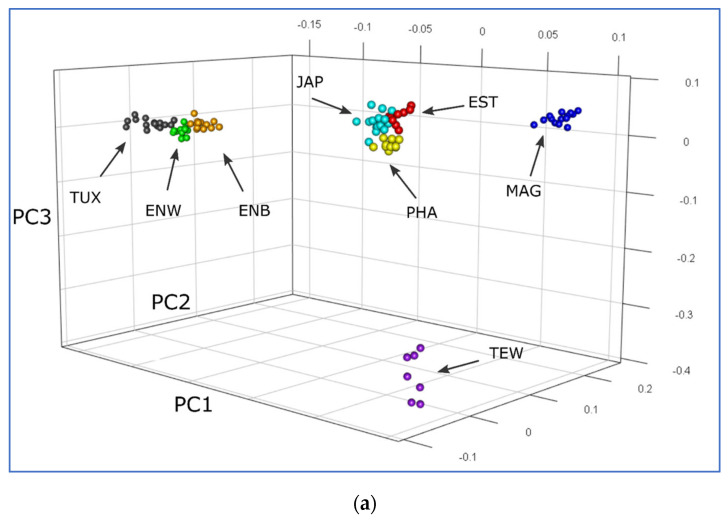

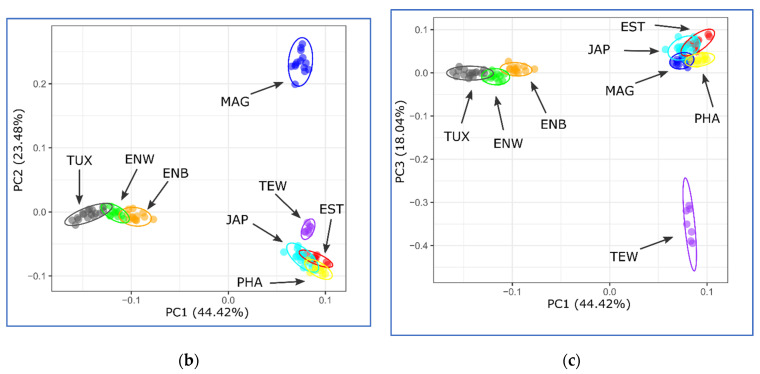
GBS-based PCA plots for the eight quail breeds studied. (**a**) View in 3D. (**b**) Plot composed in the plane of the first (*X*-axis, PC1) and second (*Y*-axis, PC2) components. (**c**) Plot drawn in the plane of the first (*X*-axis, PC1) and third (*Y*-axis, PC3) components. Quail breeds: JAP, Japanese; ENW, English White; ENB, English Black; TUX, Tuxedo; MAG, Manchurian Golden; EST, Estonian; PHA, Pharaoh; TEW, Texas White.

PCA plots for various eight quail breeds based on the individual nucleotide sequence data as obtained using the GBS method are graphically presented in Figure 2.

The first component was responsible for 44.42% of genetic variability and differentiated the cluster of ENW, ENB and TUX from the cluster of JAP, EST, PHA and TEW. The second component conformed to 23.48% of genetic differences and showed the remote position of MAG relative to the other quail breeds, i.e., demonstrated its isolation from them. In the PC1–PC3 plane, TEW differentiated from the rest, and in the PC1–PC2 plane, the MAG population was separated from the others.

**Figure 3:**
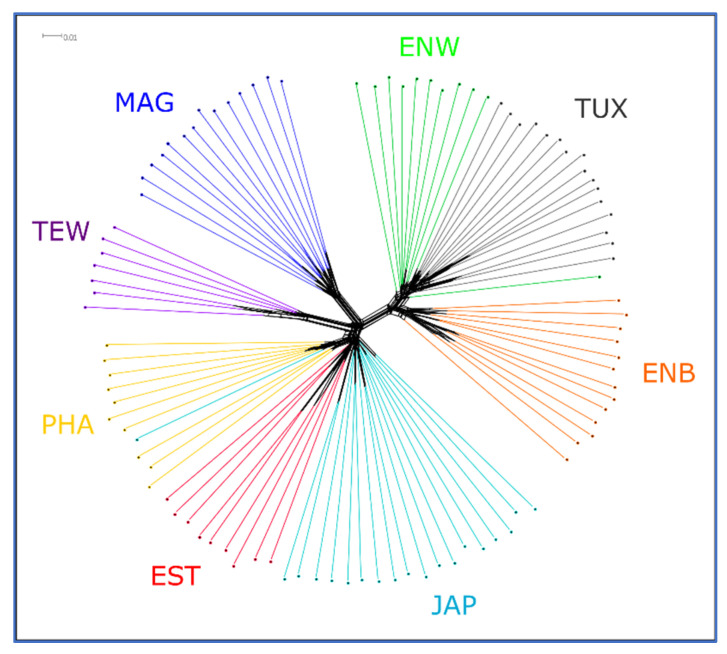
Neighbor-Net tree based on pairwise IBS distances. Quail breeds: JAP, Japanese; ENW, English White; ENB, English Black; TUX, Tuxedo; MAG, Manchurian Golden; EST, Estonian; PHA, Pharaoh; TEW, Texas White.

A Neighbor-Net tree based on the matrix of pairwise IBS distances for different quail population individuals revealed a clearcut breed differentiation judging from the distribution of individuals relative to each other in Figure 3.

**Figure 4:**
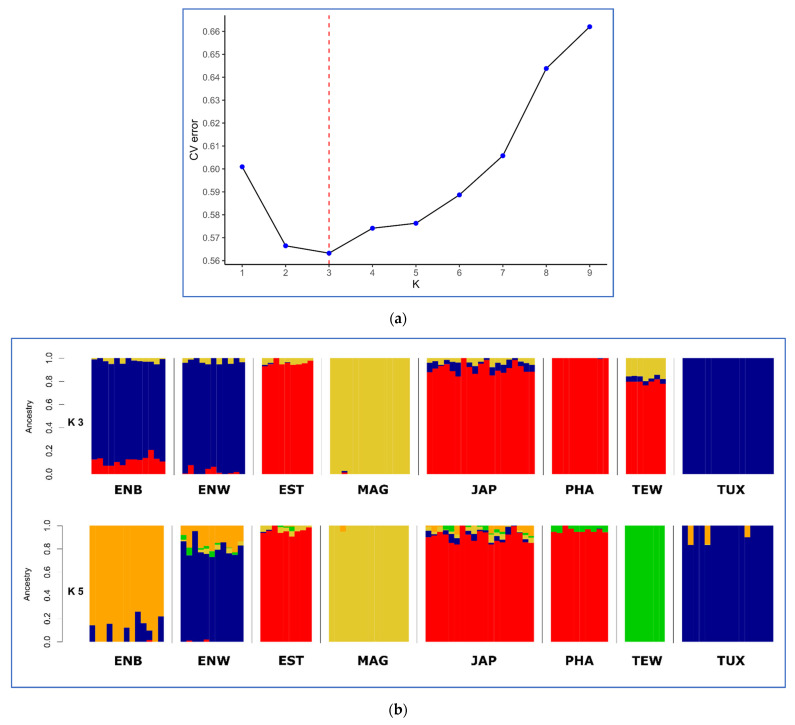

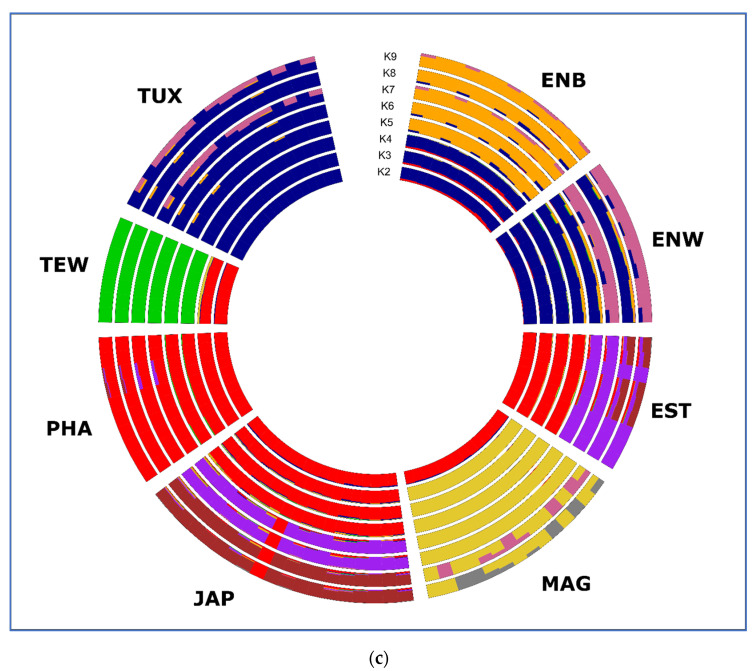
Admixture-assisted ancestry cluster analysis. (**a**) CV error calculations for different number of ancestral populations or clusters (from 1 to 9). (**b**) Horizontal view at K = 3 and 5 clusters. (**c**) Circular view for K equaling 2 to 9 clusters. Quail breeds: JAP, Japanese; ENW, English White; ENB, English Black; TUX, Tuxedo; MAG, Manchurian Golden; EST, Estonian; PHA, Pharaoh; TEW, Texas White.

During the Admixture-assisted analysis of ancestor populations and genetic impurities, calculations of the CV error for a different number of clusters (from 1 to 9) showed that the optimal number of clusters (K) was equal to 3 (Figure 4a,b). At K = 5, two groups of populations were clearly distinguished from each other as follows: (1) ENB + ENW + TUX, and (2) JAP + EST + PHA, while two single breeds, MAG and TEW, were genetically unique and distinct (Figure 4b). Clustering in the Admixture program (Figure 4c) demonstrated that MAG at K = 3 and TEW at K = 4 formed their own specific genomic pattern that was not observed in the other breeds. At the maximum tested level of clustering (K = 9), it was found that the genomic components predominantly represented in PHA were also present in EST and JAP, although this was already pronounced to a much lesser extent when K equaled 6 to 9. Shared genetic components were also observed in ENB (K = 4), ENW (K = 6 and K = 8), and TUX (K = 9).

The ENB, ENW and TUX breeds that formed a separate cluster partially overlapped each other. This was consistent with the history of the TUX descent through crossbreeding between ENW and English ENB, as well as the selection of these breeds for egg production. The formation of a joint cluster of JAP, EST, TEW and PHA breeds also conformed to the history of their origin and breeding. In particular, when creating EST, the PHA, JAP and ENW breeds were involved, and when developing TEW, the PHA breed was used.

**Figure 5:**
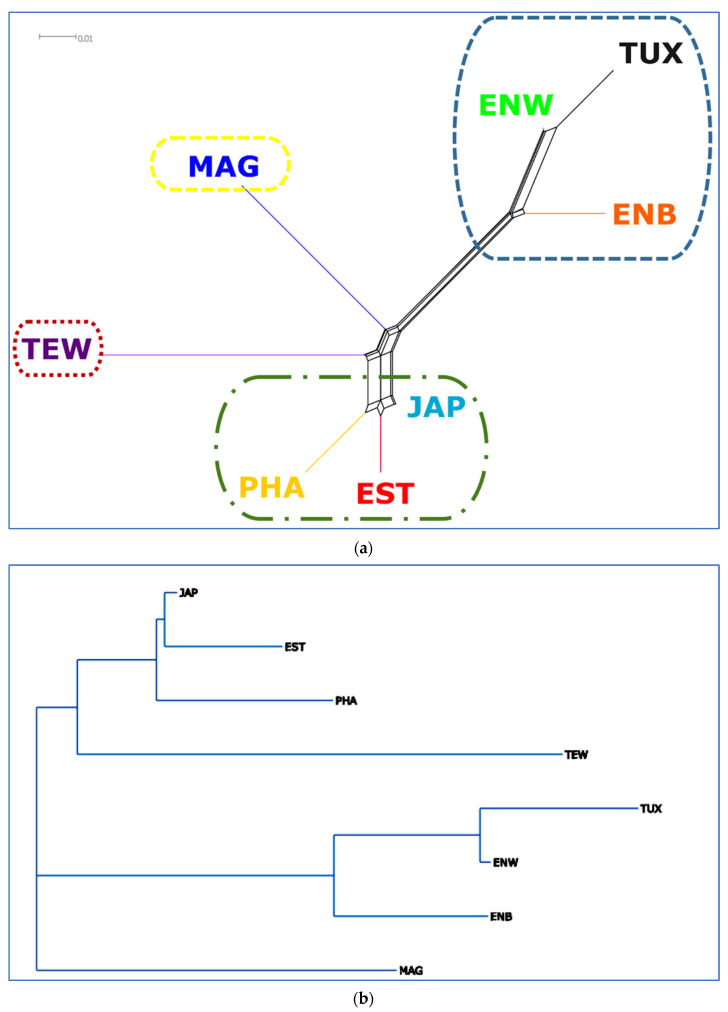
Phylogenetic trees based on *F*_ST_ genetic distances characterizing the genetic relationships between the studied quail populations. (**a**) A reconstructed Neighbor-Net network. (**b**) A Neighbor-Joining rootless hierarchical horizontal tree built with proportional edge length and using the Neighbor Joining method [55] and the online T-REX tool [54]. Quail breeds: JAP, Japanese; ENW, English White; ENB, English Black; TUX, Tuxedo; MAG, Manchurian Golden; EST, Estonian; PHA, Pharaoh; TEW, Texas White.

To visualize the genetic distances between the studied populations, a dendrogram of phylogenetic networks were constructed based on pairwise *F*_ST_ genetic distances (Supplementary Table S2) and using the Neighbor-Net algorithm (Figure 5a).

The Neighbor-Net tree based on the values of pairwise FST genetic distances showed that the PHA, EST and JAP populations formed a juncture branch and were located close to each other at the bottom edge of the graph (Figure 5a), indicating their close genetic similarity. The neighboring branch localization of ENW, ENB, as well as TUX on the reconstructed network (Figure 5a) suggested a high genetic similarity of these quail breeds, too. The positioning of the MAG population at the root of the branch suggested that the improvement of this breed occurred mainly due to the selection of purebred quails with a low contribution from other breeds. TEW was also very clearly differentiated from the other breeds, although it was included in one large cluster along with JAP, EST and PHA. The Neighbor-Joining tree had a similar topology (Figure 5b).

**Figure 6:**
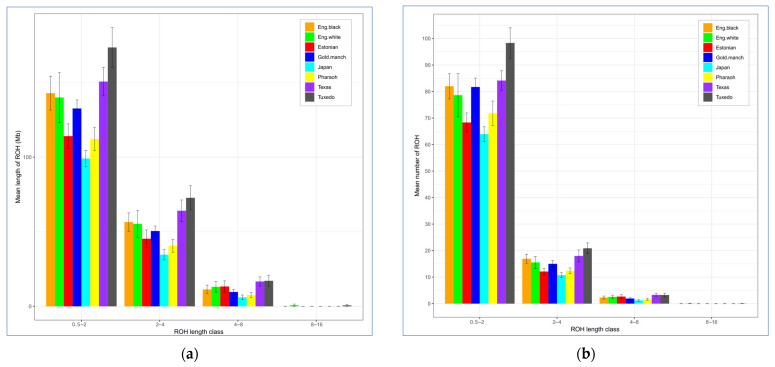
Descriptive statistics of the runs of homozygosity (ROH) according to ROH length class. (**a**) Overall mean length of ROHs (*Y*-axis) according to ROH length class (*X*-axis; 0.5–2, 2–4, 4–8 and 8–16 Mb) (**b**). Mean number of ROHs (*Y*-axis) according to ROH length class (*X*-axis; 0.5–2, 2–4, 4–8 and 8–16 Mb). Quail breeds: JAP, Japanese; ENW, English White; ENB, English Black; TUX, Tuxedo; MAG, Manchurian Golden; EST, Estonian; PHA, Pharaoh; TEW, Texas White.

Additionally, we analyzed the number and lengths of extended homozygous segments, i.e., ROHs (Table 4, Figure 6).

As can be seen from Figure 6, the greatest numbers of ROHs were within short (0.5–2 Mb) fragments. No ROHs longer than 16 Mb were found in the studied quail breeds. Those longer ROHs may be indicative of recent inbreeding events, and they were discovered in many studies in chickens (e.g., [59,60,61]). The largest number of fragments of medium length (2–4 and 4–8 Mb) was found in TEW and TUX, suggesting both ongoing breeding work aimed at consolidating desirable traits and the accumulation of homozygous fragments due to the small population size in these breeds. The smallest number and length of ROHs were observed in JAP and PHA.

**Figure 7:**
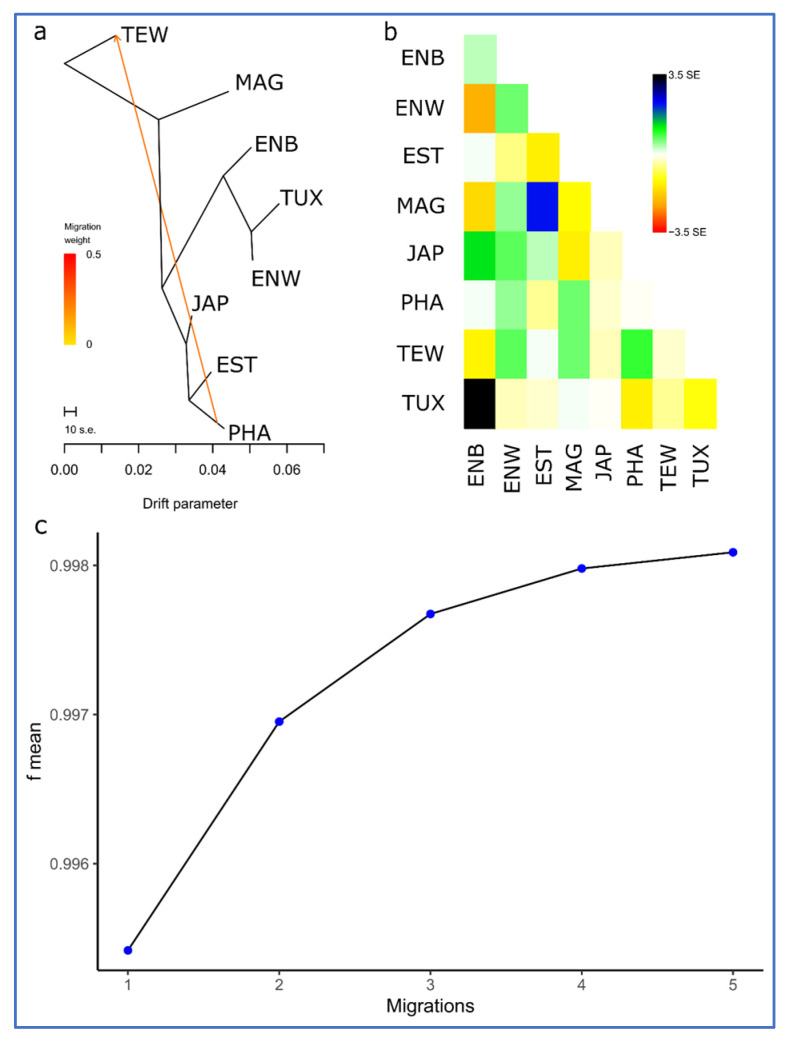
Assessed degree of divergence and the level of gene flow between the studied breeds using 30 iterations. (**a**) Rooted maximum likelihood tree with one migration event. Cut length 10 s.e. corresponds to ten times the average standard error (s.e.) estimated from the sample covariance matrix. Estimated gene flow is shown by an arrow pointing from a donor population (PHA) to a recipient one (TEW) and is colored red in proportion to the intensity of the gene flow. (**b**) Residual matrix derived from the TreeMix analysis for a single migration event expressed as the number of standard error deviations for the observations in the respective breeds. (**c**) Plot representing the proportion of variance (*f*-index) in the sample covariance matrix (¶*W*) accounted for by the model covariance matrix (*W*) as a function of the number of migration events. Quail breeds: JAP, Japanese; ENW, English White; ENB, English Black; TUX, Tuxedo; MAG, Manchurian Golden; EST, Estonian; PHA, Pharaoh; TEW, Texas White.

All variants of migration events obtained using the TreeMix program and different number of iterations (from 1 to 30) are presented in Supplementary Figure S1. The analysis of migration events revealed the presence of expectable gene flows (migrations) between the breeds. In particular, a migration from PHA to TEW was determined for the best number of iterations (29 and 30; mean SE = 0.39; Supplementary Table S3). The respective dendrogram and residual matrix (heat map) are shown in Figure 7. When using other iteration numbers, there were also eight additional observations for the gene flow events between PHA and TEW (Supplementary Figure S1). This was fully confirmed by the known fact of using PHA as one of the progenitor breeds in the creation of TEW. In addition, it can be noted on the other graphs (with different number of iterations; Supplementary Figure S1) that another migration was repeated most often (15 of 30 iteration variants) between ENB and JAP. This also fits perfectly into the origin history of ENB as a mutation of JAP. Four cases of migrations between the ancestor of ENB, ENW and TUX were also observed, which is supported by the facts that ENB and ENW are mutants of JAP and TUX stemmed from crossing ENW and ENB (Table 1).

## 4. Discussion

In the era of integrative agriculture, there is a need, in the process of monitoring, breeding and selection, to link genetic and genomic technologies to breeding regimes for economically important traits (e.g., [24,62,63,64,65]). Performance traits are highest on the list for analysis. Many crucial areas of agricultural production and research such as plant and animal breeding and trait mapping call for reliable and scalable genotyping tools. One such approach that is ideal for non-human organisms is GBS [66,67,68,69,70], which can be effective for integrating genomic and performance data. In this regard, we made an attempt to demonstrate how “congruent” interbreed patterns of genomic architecture are with those for productivity traits in quails. In our GBS study, we, for the first time, collated and compared eight breeds of domestic quail. These represent a large share (~11%) of the world gene pool of quail breeds and three purposes of their use (in terms of productive traits), and they also illustrate the evolutionary component of the selection of individuals in the process of domestication and breeding of this bird species. Having assessed the performance traits and using IPI, we quite accurately confirmed the initial (conventional) classification of these breeds that has been established in the quail breeding practice depending on their selection direction and utility type.

The revealed phylogeny pattern based on genomic data, however, had a lower congruence with the breed configuration obtained from productivity traits using IPI. We note that the egg-type breeds TUX, ENB and ENW, which form a single cluster according to genomic data (see, for example, Figure 5), are located in Table 2 on three adjacent rows (with IPI from 8.3 to 9.0). Similarly, it can be seen that the meat-type breeds PHA and TEW, also located on two adjacent rows in Table 2 (IPI = ~5), were included in one large cluster on the phylogenetic tree. No other similar patterns were observed. Apparently, genomic data reflect not only the selection direction and utility type (due to specific breeding work with breeds), but also other features of the breeds, e.g., the history of their development (namely, the original breeds and populations), as well as genetic processes occurring in individual populations (gene flow, genetic drift, random or purposeful crossbreeding, etc.). Taking into account all of the above, we can confirm that, in a general sense and to a certain degree, data on history, management and phenotype are congruent with the description of the diversity between breeds/populations (e.g., [71]).

When analyzing genetic diversity of the eight breeds, we observed the difference between inbreeding measures based on *F*_IS_ and ROH metrics. Most likely, these different estimates were a consequence of different approaches to calculating the two inbreeding indices. The F_IS_ score is calculated based on the observed and expected heterozygosities and reflects a lack (or excess) of heterozygotes, while *F*_ROH_ conforms to the proportion of homozygous regions in the genome. The latter estimate appears to be more accurate than the former one because it directly assesses genomic homozygosity. These two indicators are normally calculated and reported in similar studies (e.g., [59,61,70]) to describe different aspects of the defined genomic diversity and homozygosity.

To date, there have been a number of investigations focusing on the genetic diversity in quails. These studies were mainly related to the assessment of the genotypes of domestic and wild quails in order to characterize the genetic structure of populations in these species [22,72,73], as well as the issues of their hybridization in the wild [74,75,76]. Most of the work in this area was executed using microsatellite and mtDNA markers [72,73,74,75,76,77]. However, the use of conventional molecular markers has drawbacks and limitations in the case of both mtDNA (e.g., [78,79,80]) and microsatellite markers (e.g., [81,82]). SNP-assisted applications are more advantageous (e.g., [83,84], and as demonstrated herein). In this paper, to explore the biology of different quail species, we employed a relatively new approach through the use of SNPs obtained via whole-genome sequencing and subsequent GBS analysis. The relevance and efficiency of implementing GBS for genome-wide genotyping has also been demonstrated in other poultry, model and non-model, species, including chickens [85], ducks [86,87] and geese [88]. For instance, Grzegorczyk et al. [88] studied the genetic diversity and phylogenetic relationships of 12 Polish goose breeds using the GBS approach and identified SNPs associated with economic traits. Zhu et al. [86] developed and tested the GBS protocol for ducks, which resulted in 169,209 significant SNPs. Using GBS in ducks, SNPs and genes associated with plumage color [87] and 18 carcass traits [89] were also identified.

This approach can sometimes be employed in combination with traditional molecular markers. For example, Mathur and DeWoody [90] examined the genetic diversity of three populations of the wild Montezuma quail (*Cyrtonyx montezumae*) using whole-genome sequencing data from 74 quails. Ogada et al. [22] utilized both mtDNA and GBS analyses to investigate the genetic diversity and demographic history of wild African harlequin quail populations of Siaya County. In the current study, we demonstrated the effectiveness of SNPs, as the currently most used molecular markers, and the GBS approach for a genome-wide comparative assessment of different breeds of domestic quail. At the same time, we confirmed the high resolution and analytical power of the GBS-derived SNP scanning method for solving problems in modern genetic research, as has been previously shown in similar studies [83,91]. For instance, Weigend et al. [83] reported the evaluation of clustering accuracy for 10 chicken populations into 10 cluster groups based on microsatellite markers and SNPs. Given that the SNP data generally contained more alleles than microsatellites, these two sets of data allowed a comparison between microsatellites and SNPs as genetic markers for biodiversity research in favor of more informative genome-wide SNPs.

Using GBS analysis for the first time to analyze the genomes of different domestic quail breeds, we observed a lower genetic diversity in TUX as compared to JAP quails (*_U_H_E_* = 0.265 vs. 0.319; Table 3). A possible reason for this may be genetic drift in the TUX population that is small in size (13 animals) and has been bred for a long time as a closed population. At the same time, the higher level of genetic diversity in JAP may also reflect the crossbred origin of the individuals used in this study. Our study confirmed that GBS analysis can be considered an appropriate tool for investigating intraspecific differentiation. Therefore, the discovered similarities/differences can be used as a marker of gene flow among the studied breed samples as was shown by us here as a result of TreeMix-assisted analysis of migration edges.

The results of PCA plotting, Neighbor-Net and Admixture clustering and other related genomic analyses (Figure 3, Figure 4, Figure 5, Figure 6 and Figure 7) clearly distinguished quail breeds and utility types in line with their specific genetic origins and selection for economically important traits. This differentiation can provide important information for the collection, conservation, research and utilization of quail genetic resources as shown in other poultry breeds [26,92,93,94,95,96]. In particular, using two genetically divergent breeds, JAP and TEW (e.g., Figure 2, Figure 3 and Figure 5), we recently created a model resource F_2_ population to perform a GWAS analysis of growth dynamics in quails [97]. As a result of crossing these two contrasting breeds (slow-growing egg-type JAP and fast-growing meat-type TEW), the F_2_ population had a significant range of variability in BW and other phenotypic traits and was instrumental in identifying a series of SNPs associated with BW and a number of the respective candidate genes [97].

Collectively, based on SNP genotypes using GBS analysis, our findings illustrated phylogenetic relationships for the eight quail breeds that represented the egg type, meat type and dual purpose of use. The phylogenetic trees built on the basis of GBS data showed that the JAP, PHA and EST breeds were genetically similar to a certain degree. In addition, according to the obtained tree configuration, it can be argued that these three breeds can be especially valuable sources of genetic variability since they were close to the root of the phylogenetic tree. MAG and TEW can be classified as genetically more distant relative to the other breeds studied and to one another. The clustering of ENB, ENW and TUX into one group corresponded, in terms of their relatedness, to the historical records of the development of these breeds. Overall, we were able to show that GBS analysis is an efficient and useful instrument for elucidating genomic architecture and divergence across different quail breeds.

## 5. Conclusions

Using the GBS molecular method in the present investigation, we evaluated, for the first time, the genetic diversity of the eight quail breeds (representing about 11% of the global quail germplasm) and identified their evolutionary relationships suggesting a possible relation to their performance. In particular, the respective genetic divergence was shown for the egg (JAP, ENW, ENB, TUX and MAG), meat (TEW and PHA) and dual-purpose (EST) utility types. This study contributes to the identification of genetic differentiation and determination of relatedness between the studied breeds. In addition, it was demonstrated for the first time that the GBS analysis method is instrumental in the intra- and inter-breed assessment of genetic variation in domestic quails. The information reported here facilitates a deeper understanding of the processes of breed formation and selection in quails and can be further used to improve their economically important traits by identifying significant SNPs and candidate genes associated with these traits.

## Figures and Tables

**Table 1:** Eight quail breeds involved in this study.

Breed	Code	*n* ^1^	Origin	Refs
*Egg type*				
Japanese 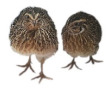	JAP	19	Japan; domesticated in Japan and China in 12th century or earlier; selected in the 1st half of the 20th century, brought to the USSR from Japan in the mid-20th century and/or from Yugoslavia in 1964	[10,11,12,13,25,26,27]
English (British) White 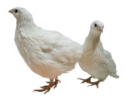	ENW	11	England; a mutant from JAP quails; imported to the USSR from Hungary in 1987	[12,13,24,27]
English (British) Black 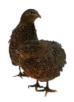	ENB	13	England; a mutant from JAP quails; imported to the USSR from Hungary in 1971	[13,27]
Tuxedo 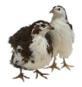	TUX	16	from crossing ENW and ENB	[12,13,27]
Manchurian (Manchu) Golden (or Golden Phoenix) 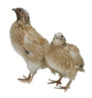	MAG	14	Marsh Farms, CA, USA, 1960s; bred by Albert Marsh as a natural mutant in a flock of brown-colored quails	[12,13,27,28,29]
*Dual purpose (or universal)*				
Estonian (or Kitevers) 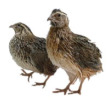	EST	9	Estonia, 1988; from crossing JAP (a Moscow line), ENW and Pharaoh	[11,13,27]
*Meat type*				
Pharaoh 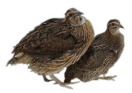	PHA	10	USA; wild-type plumage; an imported French fattening line used in this study	[12,13,26,27,30]
Texas White (or Texas Pharaoh, White Pharaoh, Snowy) 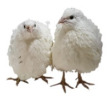	TEW	7	Texas, USA; from crossing PHA and ENW	[27,30]

^1^ *n*, number of individuals after quality control.

**Table 2:** Performance indicators ^1^ of females from the eight quail breeds studied (mean ± SD).

Breed ^2^	*n*	EN	EW	BW		IPI
				6 Weeks	6 Months	
*Egg type*						
JAP (a)	41	165.4 ± 14.7 ^a^	11.0 ± 0.9 ^a^	146.7 ± 13.6 ^a^	149.0 ± 13.2 ^a^	12.2 ± 1.2 ^a^
ENW (b)	11	134.8 ± 8.5 ^a,b^	10.2 ± 1.0 ^a,b^	157.6 ± 12.7 ^a,b^	166.6 ± 9.0 ^a,b^	8.3 ± 1.2 ^a,b^
ENB (c)	11	133.4 ± 8.0 ^a,c^	10.4 ± 0.9 ^c^	151.5 + 15.0 ^c^	159.5 ± 14.0 ^a,c^	8.8 ± 1.4 ^a,c^
TUX (d)	11	131.1 ± 7.4 ^a,d^	10.2 ± 0.9 ^a,d^	141.4 ± 10.5 ^b,d^	149.0 ± 12.6 ^b,d^	9.0 ± 0.8 ^a,d^
MAG (e)	12	147.5 ± 4.5 ^a,b,c,d,e^	10.6 ± 1.6 ^e^	168.2 ± 17.2 ^a,c,d,e^	180.1 ± 18.9 ^a,b,c,d,e^	8.8 ± 2.1 ^a,e^
*Dual purpose*						
EST (f)	18	148.9 ± 9.7 ^a,b,c,d,f^	11.8 ± 1.4 ^a,b,c,d,e,f^	248.2 ± 10.6 ^a,b,c,d,e,f^	247.3 ± 15.4 ^a,b,c,d,e,f^	7.1 ± 1.1 ^a,b,c,d,e,f^
*Meat type*						
PHA (g)	12	118.2 ± 8.2 ^a,b,c,d,e,f^	12.6 ± 0.8 ^a,b,c,d,e,f^	292.3 ± 16.2 ^a,b,c,d,e,f,g^	294.3 ± 19.5 ^a,b,c,d,e,f,g^	5.1 ± 0.6 ^a,b,c,d,e,f^
TEW (h)	23	121.4 ± 18.7 ^a,b,c,d,e,f^	12.7 ± 1.0 ^a,b,c,d,e,f^	305.5 + 21.3 ^a.b,c,d,e,f,g^	317.7 ± 25.9 ^a,b,c,d,e,f,g^	4.9 ± 1.0 ^a,b,c,d,e,f^

^1^ *n*, number of individuals; EN, egg number; EW, egg weight (g); BW, female body weight at 6 weeks and 6 months of age (g); IPI, Integral Performance Index. ^2^ Quail breeds: JAP, Japanese; ENW, English White; ENB, English Black; TUX, Tuxedo; MAG, Manchurian Golden; EST, Estonian; PHA, Pharaoh; TEW, Texas White. (a–h) Significant pairwise differences for breeds with the corresponding same superscript (*p* < 0.05); the absence of a corresponding common superscript indicates that the differences between the specific two breeds are insignificant.

**Table 3:** Characterization of the genetic diversity parameters ^1^ in the quail populations studied.

Breed ^2^	*H_O_* (M ± SE)	*H_E_* (M ± SE)	*_U_H_E_* (M ± SE)	*A_R_* (M ± SE)	*F*_IS_ [CI 95%]	*_U_F*_IS_ [Cl 95%]
*Egg type*						
JAP	0.303 ± 0.001	0.310 ± 0.001	0.319 ± 0.001	1.864 ± 0.001	0.020 [0.016; 0.024]	0.046 [0.043; 0.049]
ENW	0.281 ± 0.001	0.273 ± 0.001	0.287 ± 0.001	1.778 ± 0.002	−0.029 [−0.033; −0.025]	0.020 [0.016; 0.024]
ENB	0.282 ± 0.001	0.276 ± 0.001	0.287 ± 0.001	1.774 ± 0.002	−0.019 [−0.023; −0.015]	0.020 [0.016; 0.024]
TUX	0.265 ± 0.001	0.263 ± 0.001	0.271 ± 0.001	1.730 ± 0.002	−0.010 [−0.014; −0.006]	0.022 [0.018; 0.026]
MAG	0.286 ± 0.001	0.285 ± 0.001	0.295 ± 0.001	1.790 ± 0.002	−0.005 [−0.009; −0.001]	0.031 [0.027; 0.035]
*Dual purpose*						
EST	0.302 ± 0.001	0.295 ± 0.001	0.313 ± 0.001	1.839 ± 0.002	−0.025 [−0.030; −0.020]	0.032 [0.028; 0.036]
*Meat type*						
PHA	0.290 ± 0.001	0.286 ± 0.001	0.301 ± 0.001	1.815 ± 0.002	−0.017 [−0.021; −0.013]	0.035 [0.031; 0.039]
TEW	0.282 ± 0.002	0.264 ± 0.001	0.284 ± 0.001	1.757 ± 0.003	−0.067 [−0.072; −0.062]	0.011 [0.006; 0.016]

^1^ *H_O_*, observed heterozygosity; M, mean value; SE, standard error; *H_E_*, expected heterozygosity; *_U_H_E_*, unbiased expected heterozygosity adjusted for small samples; *A_R_*, rarefied allelic richness; *F*_IS_, inbreeding coefficient [CI 95%, range variation of *F*_IS_ coefficient at a confidence interval of 95%]; *_U_F*_IS_, unbiased inbreeding coefficient [CI 95%, range variation of *_U_F*_IS_ coefficient at a confidence interval of 95%] adjusted for small samples. ^2^ Quail breeds: JAP, Japanese; ENW, English White; ENB, English Black; TUX, Tuxedo; MAG, Manchurian Golden; EST, Estonian; PHA, Pharaoh; TEW, Texas White.

**Table 4:** Runs of homozygosity (ROHs) descriptive statistics ^1^ for the studied breeds.

Breed ^2^	ROH Length, Mb (M ± SE)			ROH No. (M ± SE)			*F*_ROH_ (M ± SE)		
	Average	Min	Max	Average	Min	Max	Average	Min	Max
*Egg type*									
JAP	99.04 ± 5.55	48.71	138.58	75.89 ± 3.38	49	104	0.119 ± 0.007	0.06	0.17
ENW	140.00 ± 16.88	8.43	216.10	96.82 ± 10.55	7	133	0.169 ± 0.020	0.01	0.26
ENB	142.92 ± 11.32	58.47	201.15	101.23 ± 6.37	54	137	0.172 ± 0.014	0.07	0.24
TUX	173.54 ± 13.36	36.79	237.00	122.50 ± 7.81	31	164	0.209 ± 0.016	0.04	0.29
MAG	132.63 ± 5.77	91.63	174.35	98.64 ± 3.93	69	121	0.160 ± 0.007	0.11	0.21
*Dual purpose*									
EST	114.11 ± 8.23	56.47	137.53	83.11 ± 4.83	49	98	0.137 ± 0.010	0.07	0.17
*Meat type*									
PHA	112.18 ± 7.86	62.22	157.96	85.80 ± 5.01	54	108	0.135 ± 0.009	0.07	0.19
TEW	150.66 ± 9.54	115.13	191.32	105.43 ± 4.74	87	122	0.181 ± 0.011	0.14	0.23

^1^ ROH No., number of ROHs in a genome; Mb, megabases; M, mean value; SE, standard error; ROH Length, overall length of ROHs in a genome; *F*_ROH_, inbreeding coefficient calculated based on ROHs. ^2^ Quail breeds: JAP, Japanese; ENW, English White; ENB, English Black; TUX, Tuxedo; MAG, Manchurian Golden; EST, Estonian; PHA, Pharaoh; TEW, Texas White.

## Data Availability

The sequence data is accessible to readers on request.

## Supplementary Materials

The following supporting information can be downloaded at: https://www.mdpi.com/article/10.3390/ani13223439/s1, Figure S1: Assessment of the degree of divergence and the level of gene flow between the studied quail breeds; Table S1: Pairwise interbreed Euclidean distances obtained for breed IPI values using the Phantasus program; Table S2: Pairwise *F*_ST_-based interbreed genetic distances; Table S3: Mean SE values according to iteration number when calculating migration events obtained using the TreeMix program.
