# Supplementary material for: Genotyping-by-Sequencing Strategy for Integrating Genomic Structure, Diversity and Performance of Various Japanese Quail (Coturnix japonica) Breeds
Source: Animals (Basel). 2023 Nov 7;13(22):3439. doi: 10.3390/ani13223439 (PMC10668688; doi:10.3390/ani13223439)
Supplement: Supplementary file 1 [file animals-13-03439-s001.zip › animals-2664280-supplementary/Suppl Table S1 revised.pdf]

Table S1. Pairwise interbreed Euclidean distances obtained for breed IPI values using the Phantasus program [53].

Breed[1]	JAP	MAG	TUX	ENB	ENW	EST	PHA	TEW	Mean
**JAP**	0.00000	3.35180	3.21940	3.44170	3.90920	5.07040	7.12120	7.29660	4.17629
**MAG**	3.35180	0.00000	0.13241	0.08990	0.55742	1.71860	3.76940	3.94480	1.69554
**TUX**	3.21940	0.13241	0.00000	0.22231	0.68984	1.85100	3.90180	4.07720	1.76175
**ENB**	3.44170	0.08990	0.22231	0.00000	0.46752	1.62870	3.67950	3.85490	1.67307
**ENW**	3.90920	0.55742	0.68984	0.46752	0.00000	1.16120	3.21200	3.38740	1.67307
**EST**	5.07040	1.71860	1.85100	1.62870	1.16120	0.00000	2.05080	2.22620	1.96336
**PHA**	7.12120	3.76940	3.90180	3.67950	3.21200	2.05080	0.00000	0.17538	2.98876
**TEW**	7.29660	3.94480	4.07720	3.85490	3.38740	2.22620	0.17538	0.00000	3.12031

[1] Quail breeds: JAP, Japanese; ENW, English White; ENB, English Black; TUX, Tuxedo; MAG, Manchurian Golden; EST, Estonian; PHA, Pharaoh; TEW, Texas White.
