# Supplementary material for: Genotyping-by-Sequencing Strategy for Integrating Genomic Structure, Diversity and Performance of Various Japanese Quail (Coturnix japonica) Breeds
Source: Animals (Basel). 2023 Nov 7;13(22):3439. doi: 10.3390/ani13223439 (PMC10668688; doi:10.3390/ani13223439)
Supplement: Supplementary file 1 [file animals-13-03439-s001.zip › animals-2664280-supplementary/Suppl Table S2.pdf]

Table S2. Pairwise F_{ST}-based interbreed genetic distances.

**Breed**	**ENB**	**ENW**	**EST**	**MAG**	**JAP**	**PHA**	**TEW**	**TUX**
**ENB**	–							
**ENW**	0.053	–						
**EST**	0.112	0.113	–					
**MAG**	0.131	0.129	0.094	–				
**JAP**	0.091	0.092	0.021	0.083	–			
**PHA**	0.119	0.121	0.045	0.107	0.035	–		
**TEW**	0.158	0.155	0.112	0.139	0.097	0.113	–	
**TUX**	0.07	0.027	0.138	0.153	0.115	0.146	0.182	–

Quail breeds: ENB, English Black; ENW, English White; EST, Estonian; MAG, Manchurian Golden; JAP, Japanese; PHA, Pharaoh; TEW, Texas White; TUX, Tuxedo.
