# Supplementary material for: Genotyping-by-Sequencing Strategy for Integrating Genomic Structure, Diversity and Performance of Various Japanese Quail (Coturnix japonica) Breeds
Source: Animals (Basel). 2023 Nov 7;13(22):3439. doi: 10.3390/ani13223439 (PMC10668688; doi:10.3390/ani13223439)
Supplement: Supplementary file 1 [file animals-13-03439-s001.zip › animals-2664280-supplementary/Suppl Table S3.pdf]

Table S3. Mean SE values by iteration number when calculating migration events obtained using the TreeMix program.

**Iteration number**	**Mean SE value**
1	0.58620785554332
2	0.58620785554332
3	0.607671354182819
4	0.607671354182819
5	0.557669377893411
6	0.557669377893411
7	0.584110074448439
8	0.563082101675655
9	0.563082101675655
10	0.5264392604876
11	0.5264392604876
12	0.588408184911085
13	0.588408184911085
14	0.577102810564631
15	0.577102810564631
16	0.587515502950406
17	0.587515502950406
18	0.64992328095135
19	0.64992328095135
20	0.530395631537119
21	0.530395631537119
22	0.468369771460307
23	0.518952646750049
24	0.518952646750049
25	0.571793783630506
26	0.571793783630506
27	0.461874426436555
28	0.461874426436555
29	0.390936345587229
30	0.390936345587229
