# Supplementary material for: Genotyping-by-Sequencing Strategy for Integrating Genomic Structure, Diversity and Performance of Various Japanese Quail (Coturnix japonica) Breeds
Source: Animals (Basel). 2023 Nov 7;13(22):3439. doi: 10.3390/ani13223439 (PMC10668688; doi:10.3390/ani13223439)
Supplement: Supplementary file 1 [file animals-13-03439-s001.zip › animals-2664280-supplementary/Supplementary Figure S1.pdf]

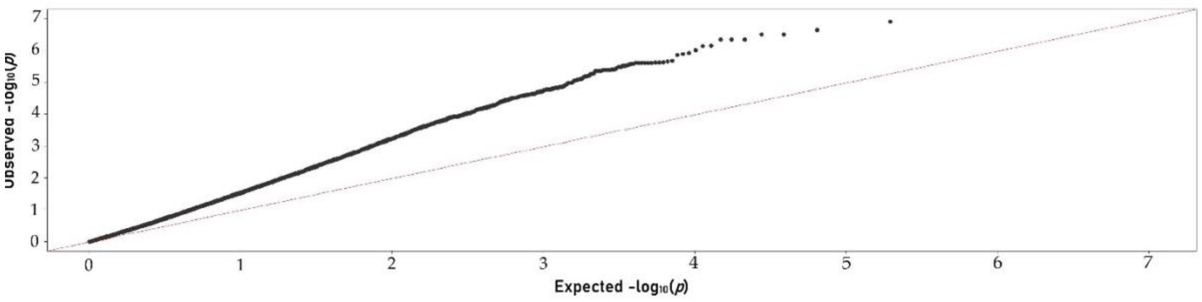
Observed -log10(p)
Expected -log10(p)
0 1 2 3 4 5 6 7

A

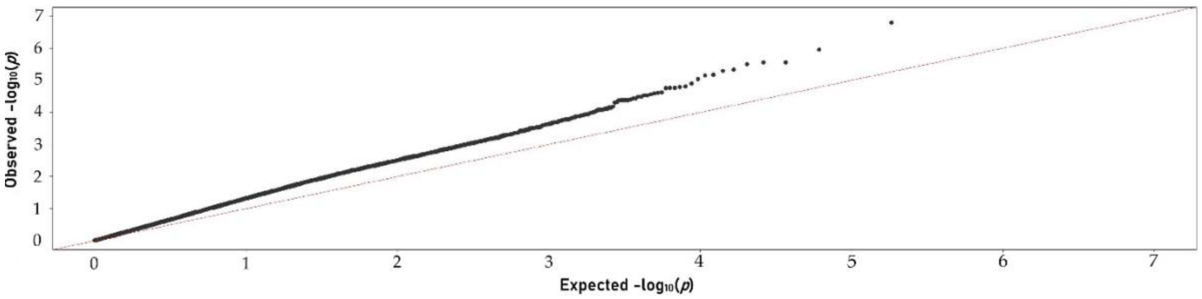
Observed -log10(p)
Expected -log10(p)
0 1 2 3 4 5 6 7

B

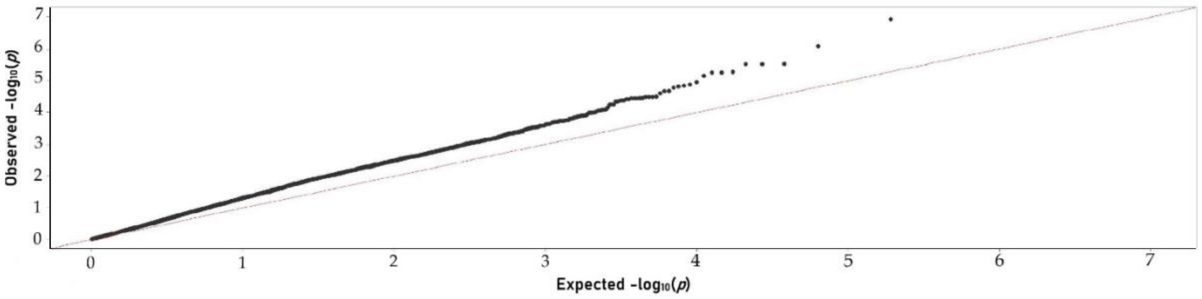
Observed -log10(p)
Expected -log10(p)
0 1 2 3 4 5 6 7

C

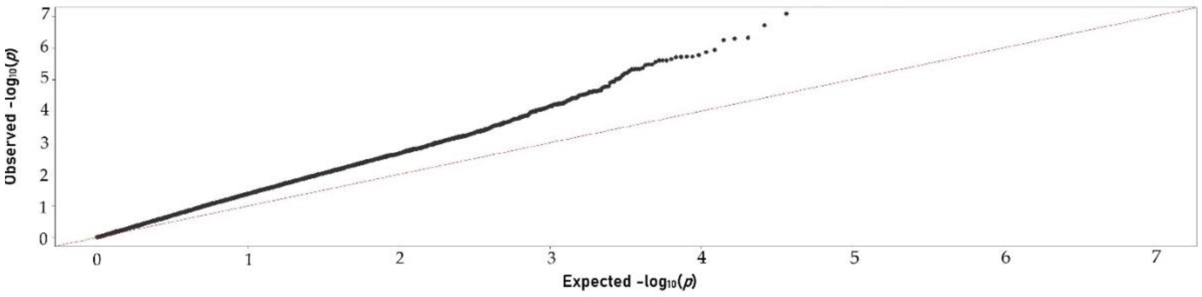
Observed -log10(p)
Expected -log10(p)
0 1 2 3 4 5 6 7

D

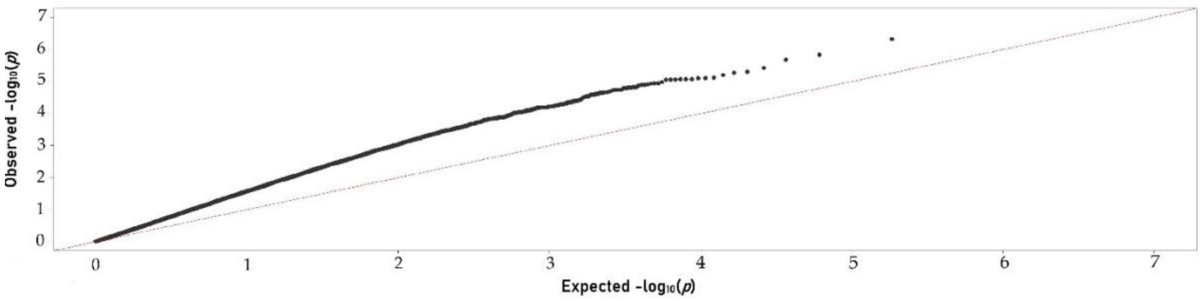
Observed -log10(p)
Expected -log10(p)
0 1 2 3 4 5 6 7

E

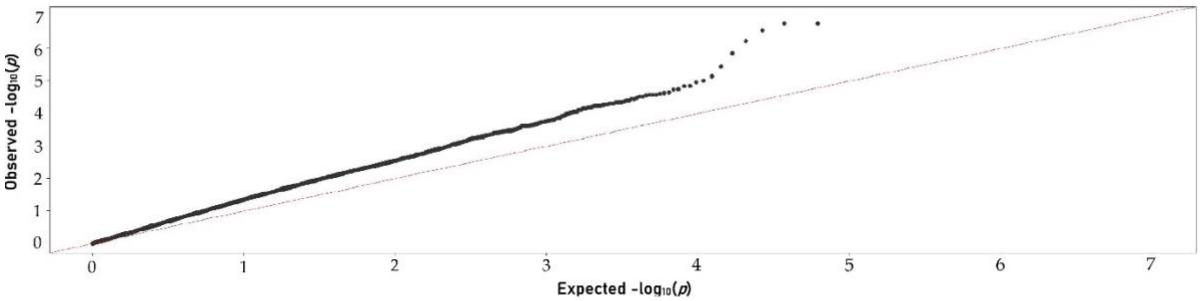

F

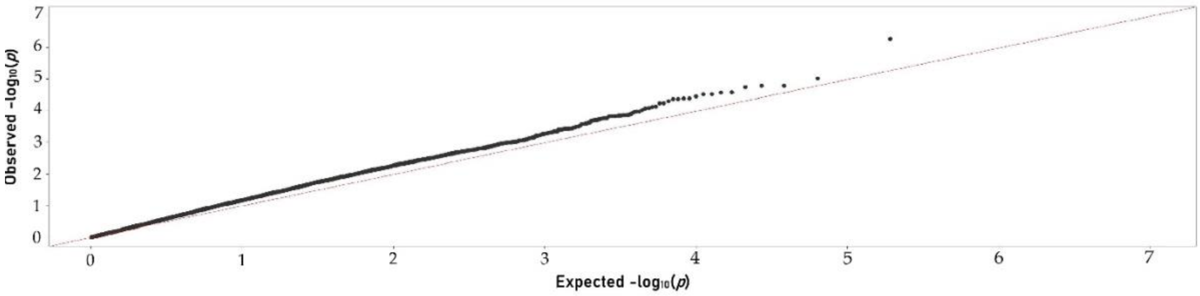

G

Figure S1. Quantile–quantile (Q–Q) plots of the GWAS results for the studied growth and meat production traits: (**A**) body weight at 1 day of age, (**B**) body weight at 56 days of age, (**C**) average daily body weight gain, (**D**) dressed carcass weight, (**E**) breast weight, (**F**) thigh weight, and (**G**) drumstick weight. Q–Q plots represented quantiles of the probability distribution of expected and observed deviations from the normal distribution for significance values (−log10 (*p*)).
